# Granulomatous Amyloid Angiopathy in a Patient With Persistent Headache

**DOI:** 10.7759/cureus.55071

**Published:** 2024-02-27

**Authors:** Richa Tikaria, Maham A Khan, Howard Chang

**Affiliations:** 1 Internal Medicine, Michigan State University, East Lansing, USA; 2 Pathology, Sparrow/Michigan State University (MSU) Neurology and Ophthalmology, Lansing, USA

**Keywords:** cerebrospinal fluid studies, s: biomarkers, age-related disease, hemorrhage - cerebral, cerebral amyloid angiopathy

## Abstract

Cerebral amyloid angiopathy (CAA) is a type of cerebrovascular disorder characterized by the accumulation of amyloid beta peptide within the walls of small to medium-sized blood vessels in the brain and leptomeninges. This can cause a variety of symptoms, depending on the location and extent of the deposits. Common presentations of amyloid angiopathy include cognitive decline, memory loss, headaches, vision changes, stroke-like symptoms, and seizures. In some cases, there may be no noticeable symptoms. A 78-year-old female was admitted for ongoing evaluation of a persistent headache after her primary care physician (PCP) ordered outpatient magnetic resonance imaging (MRI) that showed findings concerning metastatic tumors versus infectious processes. She underwent a right temporal lobe biopsy, which confirmed the diagnosis of granulomatous amyloid angiopathy.

## Introduction

Amyloid angiopathy is a common condition that affects a significant portion of the elderly population. Based on neuropathological examination, the prevalence of moderate-to-severe Cerebral amyloid angiopathy (CAA) in Alzheimer's disease (AD) is 48%. [1] CAA is the most common cause of lobar intracerebral hemorrhage (ICH) in the elderly and results from abnormal deposition of β-amyloid protein in the walls of the brain and leptomeninges vessels [2-3]. A subgroup of CAA patients may experience spontaneous inflammation, known as CAA-related inflammation (CAA-RI), presenting with symptoms like headaches, seizures, focal neurological deficits, and subacute cognitive decline. [4] This disease can be diagnosed by various modalities, such as MRI, positron emission tomography (PET), cerebrospinal fluid (CSF) studies, or biopsy [5]. To diagnose it early with noninvasive methods, a high level of suspicion is required.

Treatment for CAA-RI typically involves aggressive immunosuppression, predominantly with high-dose intravenous steroids, followed by a prolonged taper (a minimum of six months). In some cases, providers may incorporate cyclophosphamide, especially for Aβ-Related Angiitis (ABRA). [4] Timely management of amyloid angiopathy is important in order to prevent progression and potential complications.

## Case presentation

**Figure 1:**
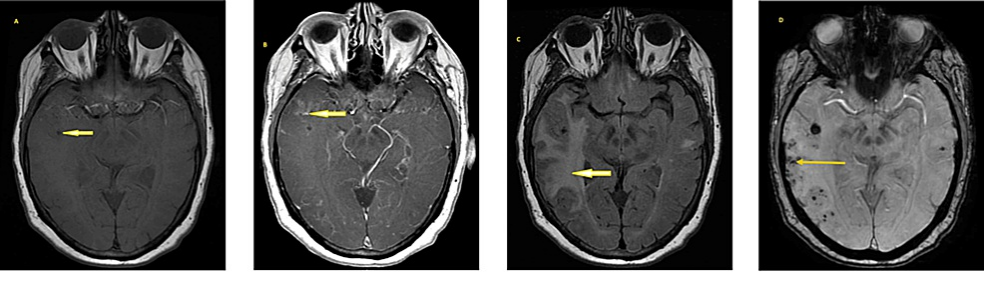
MRI brain (A) T1 pre-contrast, (B) T1 post-contrast, (C) T2 flair, (D) SWAN

A 78-year-old female with a medical history of hyperlipidemia, type 2 diabetes mellitus, endometrial carcinoma status post-hysterectomy, and bilateral salpingo-oophorectomy nine years prior presented to the emergency department in October 2023 after referral by her PCP. Six months prior, she first noticed a sharp pain localized to the right side of her head, along with the sensation of facial weakness on the same side. She denied noticing any facial drooping. However, given the nature of her symptoms, she was concerned about a stroke and went to the nearest emergency department. She had elevated blood pressure and underwent a computed tomography (CT) of the brain that was unremarkable. During a follow-up visit with her primary care physician (PCP) after that emergency department (ED) visit, she was recommended for an evaluation by neurology and an MRI of the brain. There was a delay in completing the imaging due to the decline in her spouse’s health. Her daughter reported that during this time, the patient had also been experiencing difficulty expressing herself and forgetfulness. The MRI (Figure 1) demonstrated multifocal areas of vasogenic edema and petechial hemorrhages involving the right parietal and bilateral temporal lobes, a localized mass effect, and a midline shift to the left. Along with the regions of vasogenic edema, the medical team also observed diffuse nodular leptomeningeal enhancement. These findings were atypical of infracts and raised concern for metastatic disease and infectious or inflammatory encephalitis. She was advised to come to the ED.

Her initial vitals were consistent with a hypertensive emergency, with a blood pressure reading of 208/81 mm Hg. Her physical exam, including a neurological exam, was completely unremarkable. She was admitted to the neuro-critical care service for blood pressure control with a nicardipine drip, and neurosurgery was consulted. She underwent a lumbar puncture (LP), and studies were sent for cytology, culture, protein, glucose, cell count, cytomegalovirus (CMV), West Nile virus, Venereal Disease Research Laboratory (VDRL), John Cunningham virus (JC virus), DNA, Varicella-Zoster virus (VZV), Herpes simplex, cryptococcal antigen, autoimmune encephalopathy panel, and flow cytometry to evaluate for infection, inflammatory, and neoplastic etiologies (Table 1).

**Table 1:** Cerebrospinal fluid tests and their results ACE: angiotensin-converting enzyme, CMV: cytomegalovirus, VDRL: venereal disease research laboratory, CASPR2: contactin-associated protein-like 2, CBA: cell-binding assay, DPPX: dipeptidyl-peptidase-like protein, GABA: gamma-aminobutyric acid receptor, GAD65: glutamic acid decarboxylase 65-kilodalton isoform, GFAP: glial fibrillary acidic protein, AMPA-R Ab: alpha-amino-3-hydroxy-5-Methyl-4-isoxazole Propionic Acid receptor, VZV: Varicella Zoster virus, JC virus: John Cunningham virus, mGluR1: metabotropic glutamate receptor 1, NMDA-R: N-methyl-d-aspartate-receptor

Test name	Results
Protein	110 mg/dl (normal range: 15 – 45)
Glucose	68 mg/dl (40–80)
ACE	<1.5 U/L (0.0–3.1)
Cell count	RBC 3/cu mm, WBC 1/cu mm
Cytology	Negative for malignancy
Bacterial culture, and Gram stain	No growth, no WBCs, organisms observed
Fungal culture	No growth
CMV DNA qualitative, PCR	Not detected
West Nile virus IgG, IgM	Negative
VDRL	Non-reactive
VZV by PCR	Not detected
JC virus DNA, PCR	Negative
Herpes simplex I/II by PCR	Not detected
Cryptococcal antigen, fluid	Negative
AMPA-R Ab cell-binding assay, CSF	Negative
Amphiphysin Ab	Negative (<1:2)
Anti-glial nuclear Ab, type 1	Negative (<1:2)
Anti-neuronal nuclear Ab, type 1	Negative (<1:2)
Anti-neuronal nuclear Ab, type 2	Negative (<1:2)
Anti-neuronal nuclear Ab, type 3	Negative (<1:2)
CASPR2-IgG CBA	Negative
Contactin-associated protein-like 5 (CRMP-5)-IgG	Negative (<1:2)
DPPX Ab immunofixation	Negative
GABA Ab cell binding assay	Negative
GAD65 Ab assay	Negative (<=0.02nmol/L)
GFAP immunofixation assay	Negative
Anti-leucine-rich glioma inactivated 1 (LGI1) IgG cell-binding assay	Negative
mGluR1 Ab immunofixation assay	Negative
NMDA-R Ab cell binding assay	Negative
Purkinje cell cytoplasmic Ab type titer	Negative (<1:2)
Purkinje cell cytoplasmic Ab type 1	Negative (<1:2)
Purkinje cell cytoplasmic Ab type 2	Negative (<1:2)
Leukocyte/lymphocyte immunophenotyping by flow cytometry of CSF	Paucicellular specimen with a few T-cells, and no B cells

To investigate the possibility of malignancy, a CT of the chest, abdomen, and pelvis, along with tumor markers (CEA and CA 125), was ordered, but the results came back negative. She also underwent an MRI of the thoracic and lumbar spine with and without contrast that demonstrated age-related changes but no acute process. Her blood pressure was difficult to control throughout her admission, and she was ultimately discharged on Norvasc 10 mg and Lisinopril 30 mg. She was advised to follow up as an outpatient with neurosurgery for a brain biopsy while her spinal fluid labs were in process.

**Figure 2:**
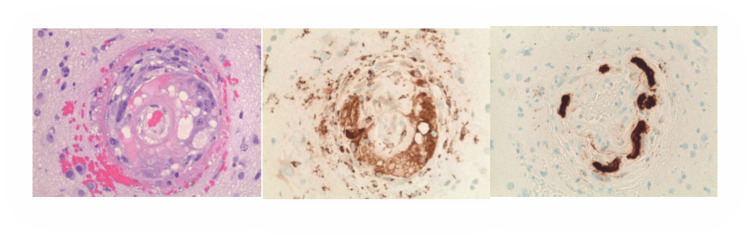
(A) The brain biopsy haematoxylin and eosin (H-E stained) from the right temporal cortex shows an inflammed cerebral vessel surrounded by multinucleated giant cells. Micrographs taken with a 20× objective lens (original magnifications: 200×). (B) Immunohistochemistry reaction shows that the multinucleated cells are positive for CD68, a marker for macrophages. Micrographs taken with a 20× objective lens (original magnifications: 200×). (C) Immunohistochemistry reaction shows vessel wall is positive for beta-amyoid. Micrographs taken with a 20× objective lens (original magnifications: 200×).

Upon follow-up, the patient reported the resolution of her headaches, although memory issues persisted. Additionally, her blood pressure was measured at 128/80. The brain biopsy included sampling from the right temporal dura, arachnoid, and brain parenchyma. The biopsy showed granulomatous amyloid angiopathy/angiitis (Figure 2). There was no evidence of malignancy. 

## Discussion

This case of an elderly patient presenting with an intracranial hemorrhage highlights the importance of considering CAA as a potential cause. CAA is characterized by the presence of amyloid beta protein within cortical and leptomeningeal blood vessel walls and is more commonly seen in individuals with Alzheimer’s disease. While the incidence of CAA in the United States is not well defined, some estimates suggest that CAA may be present in up to 30% of elderly individuals and up to 50% of individuals over the age of 85 [6].

CAA is also known to be a risk factor for cerebral hemorrhage and is estimated to be responsible for about 10-20% of all spontaneous ICHs in elderly individuals [7]. The Boston criteria, comprising combined clinical, imaging, and pathological parameters, were proposed to standardize the diagnosis of CAA. However, over half of the patients with CAA are missed by these criteria [8].

Recent advances in magnetic resonance imaging and cerebrospinal fluid biomarker analysis have furthered our understanding of CAA. Currently, there is no specific treatment for symptomatic CAA other than systemic treatment and the avoidance of anticoagulants due to the risk of hemorrhage. The clinical impact on cognition is also a concern, as is the possibility of transient focal TIAs. The use of statins in CAA is controversial, and immunotherapy trials are in process but not yet approved for clinical use [6].

In our patient’s case, the initial MRI imaging was interpreted as concerning metastatic disease or an inflammatory, infectious encephalitis, or vasculitis. Amyloid angiopathy was not high on the differential list initially considered. However, a cerebrospinal fluid sample collected from the patient could have been sent for amyloid beta testing, a less invasive way to confirm the diagnosis of CAA.

## Conclusions

Common presentations of amyloid angiopathy include cognitive decline and memory loss, which are often seen in various forms of age-related dementia. We are only recently beginning to learn about the impact that CAA has on cognition. CAA should be considered a potential cause in elderly patients presenting with intracranial hemorrhage, particularly those with underlying dementia. Further research is needed to improve the diagnosis and management of CAA, and the use of biomarkers for the diagnosis of CAA should be considered in the future.
